# The effects of pyridaben pesticide on the DNA integrity of sperms and early in vitro embryonic development in mice

**Published:** 2013-08

**Authors:** Ghodrat Ebadi Manas, Shapour Hasanzadeh, Golamreza Najafi, Kazem Parivar, Parichehr Yaghmaei

**Affiliations:** 1 *Department of Biology, Science and Research Branch, Islamic Azad University, Tehran, Iran.*; 2 *Department of Basic Veterinary Sciences, Histology and Embryology Sections, Faculty of Veterinary Medicine, Urmia University, Urmia, Iran.*

**Keywords:** *Pyridaben*, *Invitrofertilization*, *DNAdamage*, *Mice*

## Abstract

**Background:** Pyridaben, a pyridazinone derivative, is a new acaricide and insecticide for control of mites and some insects such as white flies, aphids and thrips.

**Objective:** This study was designed to elucidate how pyridaben can affect the sperms' morphological parameters, its DNA integrity, and to estimate the effect of various quantities of pyridaben on in vitro fertilization rate.

**Materials and Methods:** In this study, 80 adult male Balb/C strain mice were used. Animals were divided into control and two test groups. Control group received distilled water. The test group was divided into two subgroups, viz, high dose (212 mg/kg/day) and low dose (53 mg/kg/day) and they received the pyridaben, orally for duration of 45 days. The spermatozoa were obtained from caudae epididymides on day 45 in all groups. Sperm viability, protamin compression (nuclear maturity), DNA double-strand breaks, and in vitro fertilizing (IVF) ability were examined.

**Results:** The pyridaben treatment provoked a significant decrease in sperm population and viability in epididymides. The data obtained from this experiment revealed that, the pyridaben brings about negative impact on the sperm maturation and DNA integrity in a time-dependent manner, which consequently caused a significant (p<0.05) reduction in IVF capability. Embryo developing arrest was significantly (p<0.05) higher in treated than the control group.

**Conclusion:** Theses results confirmed that, the pyridaben is able to induce DNA damage and chromatin abnormalities in spermatozoa which were evident by low IVF rate.

This article extracted from Ph.D. thesis. (Ghodrat Ebadi Mans)

## Introduction

Approximately 15-20% couples in their reproductive age suffering from infertility, out of which the male infertility contributes for about half of all this population (1). Among the variety of causes, environmental factors such as chemical agents and drugs seem to be the most important factor of infertility (2). 

In recent years, reduction in fertility in male due to drop in sperm count and consequently decrease in reproductive capacity in human population has received enhanced interest. With reference to a repot among men without a history of infertility, the mean sperm count declined from 113 million/ml in 1940 to 66 million/ml in 1990 (1). Furthermore, a significant decrease in mean seminal volume from 3.40 ml to 2.75 ml was reported during the same period, indicating an even more pronounced decrease in the total sperm count (1). 

A more recent study confirmed the drop in sperm counts over the past 20 years and, in addition, a decline in sperm motility as well as in the percentage of morphologically normal sperm (3). Pyridaben is the most recently introduced chemical for the control of all developmental stages of the spider mites (4, 5). Pyridaben has been studied for acute toxicity in different species. Major clinical signs observed were decreased spontaneous motor activity, abnormal gait, arched back posture, eye closing, piolerection and bradypnea (6). Pyridaben is selective and stoichmetric inhibitor of complex I in the inner membrane of mitochondria (7). 

When complex I is activated, mitochondrial nitric oxide synthase (mtNOS) shows considerable enzymatic activity and generate nitric oxide (NO) whereas inhabitation of complex I by pyridaben guides the mtNOS to lose ties with NO producing activity and to become a O_2_ source (8, 9). Pyridaben was also highly toxic to midbrain organotyoic slice and suggest the need for evaluation of the potential for these compounds to damage the dopaminergic system in animal and to determine whether there is significant human exposure to these pesticides (10). 

Pyridaben is twice more potent than rotenone at inhibiting mitochondrial respiration. Pyridaben is a competitive inhibitor of DHR (dihydrorotenone) binding, indicating positive cooperativity (11, 12). The greater hydrophobicity of pyridaben may result in concentration of pyridaben in the membrane where the enzyme complex is located, resulting in enhanced inhibitory potency, therefore high potency of pyridaben causing cell death. Pyridaben causes significantly greater oxidative damage than a similar dose of rotenone (13). 

Partial inhibition of complex I by pyridaben is sufficient to produce significant increase in reactive oxygen species (ROS) production (14-17). Pyridaben is a widely used miticide, binding to the PSST subunit of complex I and inhibits electron flow (18). Pyridaben has not been fully evaluated for its possible immunotoxic effect, whereas known that, it is a potent inducer of apoptosis in ST486 cells and in EW36 cells in combination with heat stress (19). In addition, inhabitation of electron flow at complexes I can generate excess ROS, and an oxidative stress condition, contributing to mitochondrial dysfunction (20-22). 

It has been indicated that, pyridaben miticide inhabitation of NADH: ubiquinone oxidoreductase activity leading to the level of induced ornithine decarboxylase activity which causes anti-proliferative effect and thus anticancer action (23). The aim of this study was to evaluate the probable simultaneous effect of pyridaben on semen quality, sperms' DNA damages and in vitro fertilizing ability of sperms on time depending manner. 

## Materials and methods

**Animals and treatment groups**

This study was experimented on** e**ighty adult Balb/C strain mice of 10 weeks age and 25±3g weight, maintained in laboratory animal housing facilities under controlled light conditions 12 hour light and 12 hour dark and temperature between 20-23^o^C. The animals were fed on standard mice pellet and watered ad libitum. In this study all experiments were conducted in accordance with the principles and procedures outlined by Urmia University guidance of ethical committee for research on laboratory animals. 

The animals were divided in to control and test groups. The test subgroups nominated upon the dosage of pyridaben in the study as high dose (212 mg/kg) and low dose (53 mg/kg). The mice in control group received 0.2 ml distilled water. The high dose and low dose experiment groups received pyridaben at rate of 212mg/kg and 53 mg/kg through oral route (by gavage) respectively for the duration of 45 days. 

**Sperm collection**

After running of treatment period, each mouse was sacrificed by decapitation according to recommendation of the institutional ethical committee. Spermatozoa were obtained from cauda epididimis under a 20-time magnification provided by a stereo zoom microscope (model TL2, Olympus Co., Tokyo, Japan). Both cauda epididimides of each mouse was trimmed and minced in 1 ml HTF+4 mg/ml BSA medium pre-warmed to 37^o^C. After 20 minute incubation at 37^o^C in an atmosphere of 5% CO_2_ and the grinded epididymal tissue was separated from the released spermatozoa.

**Epididymal sperm count and viability** **assay**

The cauda epididymis sperm reserves were determined using the standard hemocytometry (1). Sperm viability was analyzed using eosin- nigrosin staining technique. Ten microliters of each semen sample were placed on slide and stained with ten microliters of eosin-nigrosin. The live (non-stained), dead (red-stained heads), abnormal and morphologically immature sperms were evaluated (24). 

**Sperm chromatin integrity assay**

Acidic aniline blue staining was used to detect chromatin defects of sperm nuclei related to their nucleoprotein content as associated with DNA. The Aniline blue (Ab) staining specifically reacts with lysine residues in nuclear histones and reveals differences in the basic nuclear protein composition of the sperm. Histone-rich nuclei of immature sperm are rich in lysine and will consequently take up the blue stain. On the other hand, protamine rich nuclei of mature sperm are rich in arginine and cysteine and contain relatively low levels of lysine, which means they will not be stained by Ab (25). A drop of semen was spread on the glass slides and allowed to air-dry. All the smears were fixed in 3% buffered glutaraldehyde for 30 min. The slides were then stained with 5% aqueous aniline blue and mixed with 4% acetic acid (pH= 3.5) for 7 min then evaluated under light microscope. 

**DNA double strand breaks assay**

A drop of semen was spread on the glass slides and allowed to air-dry. All the smears were fixed in methanol/acetic acid (3:1). The slides were then stained with 19% acridine-orange solution in phosphate citrate for 10 min in each slide. The sperms were evaluated with fluorescence microscope (Model GS7, Nikon, Japan) with a 100 oil immersion objective lens. Three types of staining patterns were identified; green sperms (double-stranded DNA), yellow and red sperms (single-stranded DNA) (26).

**Collection of ovulated oocytes and in vitro fertilization**

Superovulation was induced in 6-7 weeks old female mice by intraperitoneal injection of 10 IU pregnant mare serum gonadotropin (PMSG sigma, G4877) followed by intraperitoneal injection of 10 IU HCG (Sigma, C1063) 48 h later. At 12-14 hours post HCG injection, female mice were scarified by cervical dislocation. The oviducts were removed and the ampulla portion was put into a plastic dish containing HTF+ 4mg/kg BSA medium. The oocytes in cumulus masses were dissected out of the oviducts and introduced into the HTF+ 4mg/kg BSA medium. Microdrops of fertile sperm (1×10^6^ sperm/ml) in HTF+ 4mg/kg BSA were prepared, and 10 to 15 oocytes were placed into each sperm microdrop (150 µl). 

The fertilization process was performed for four to six hours incubation at 37^o^C under 5% CO_2_. The cumulus cell free fertilized oocytes were transferred to fresh drops of HTF+ 4mg/kg BSA medium for culture of embryos. All of the medium droplets were covered with mineral oil (sigma M8410) and fertilized oocytes were evaluated by appearance of the pronuclei and polar bodies under the inverted microscope with magnification of 200**ₓ**. After IVF, zygotes were washed 3 times with potassium simplex optimized medium (KSOM) and then transferred into fresh KSOM, cultured for an additional 5 days by incubation at 37^o^C under 5% CO_2_. 24 hours after the zygotes culture, the two cell embryos rate as well as in vitro embryonic development were evaluated, during 5 days under phase-contrast microscopy to blastocyst stage. 

**Statistical analysis**

Statistical analyses were performed on all data using ANOVA, by SPSS software version 16.0. All values were expressed as the mean±SE and p<0.05 was considered to be statistically significant. 

## Results

**Sperm count and morphology**

**Figure1:**
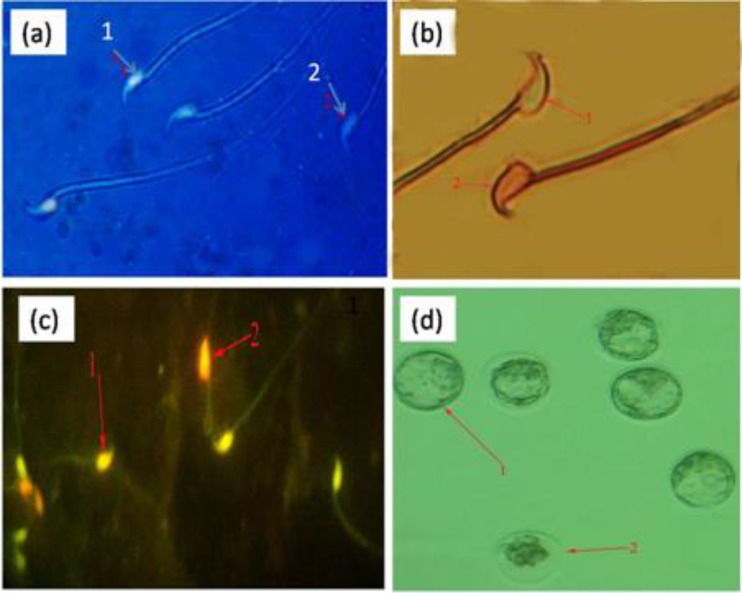
(a) aniline-blue staining for spermatozoa indicate that 1- mature sperm with light nucleus and 2- immature sperm with blue nuclei. (b) eosin- negrosin staining for spermatozoa indicate that 1- intensive sperm with light nuclei and 2- dead sperm with red nucleus (×1000). (c) acridine -orange staining for spermatozoa indicate that 1- mature sperm with light nuclei and 2- the number of sperms with double-strand DNA breaks (×1000). (d) 1- blastocyst after IVF in control group with 2- remarkably fragmented embryo (invert microscope ×1000).

Our results revealed that, in test group which received high dose of pyridaben, in comparison to the controls, the sperm count was reduced significantly (p<0.05) after 45 days of administration (Table I). Aniline blue staining for sperm nucleus maturity revealed that, the ratio of nuclear immature sperms (light stained nucleus) was increased remarkably in pyridaben groups (low and high dose) in comparison to other test groups (Figure 1a). Furthermore the sperms with cytoplasmic droplets were observed as immature sperm. Our observations demonstrated that, the number of sperms with cytoplasmic droplets increased significantly (p<0.05) in the 45^th^ day in both pyridaben treated groups.

In the eosin-negrosin staining, those sperms with stained heads were considered as dead. Intensive sperm mortalities were observed in pyridaben groups mice with dose dependant mode (Figure 1b). It is to notify that, the severities of sperm parameters anomalies were more in high dose than the low dose exposed animals. The data for sperm count and morphology is presented in table I. The acridine-orange staining showed that, the number of sperms with double-strand DNA breaks are significantly (p<0.05) intensified in the pyridaben exposed groups than the controls and this was more enhanced after 45 days exposure (Figure1c). The numerical data for sperm DNA fragmentation and breaks is presented in table I. 

**The pyridaben influences the rate of fertilized oocytes**

The in vitro fertilizations of oocytes by sperms which were collected from the pyridaben exposed groups animals were remarkably lower than the control group. The most considerable occurrence was that, in most cases the progression of 2-cell embryos in to four and/or more cells embryos in pyridaben exposed groups stopped. Moreover, the majority of divisions were arrested in samples from the pyridaben exposed groups. By comparing the values for rate of fertilized oocytes, 2 cells and blastocytes between all of the groups indicated that the highest value belongs to the oocytes that exposed to sperms from the high dose of pyridaben group in the 45^th^ day (Figure 1d). The data for in vitro fertilization rates is presented in table II.

**Table I:** Comparative parameters of sperm in control and experimental groups (Mean ± SE)

**Different parameters**	**Control**	**Low dose**	**High dose**
Sperm count (10^6^)	51.33 ± 1.45 ^a^	36.67 ± 4.66^ a^	24.66 ± 3.48 ^b^
Sperm viability (eosin-nigrosin) (%)	90.66 ± 1.45 ^a^	71.00 ± 3.46^b^	60.33 ± 2.72 ^b^
Immature sperm (aniline blue) (%)	8.0 ± 0.58 ^a^	14.66 ± 0.88 ^b^	24.00 ± 4.04 ^b^
DNA double strand sperm (acridine orange) (%)	9.67 ± 0.88^a^	16.00 ± 0.57^b^	26.00 ± 2.08^b^

The different parameters in the control and test groups are analyzed through Tukey’s test. In each row the identical superscript indicates non- significant (P>0.05) difference between parameters.

**Table II:** Comparative parameters of embryos in control and experimental groups (Mean ± SE)

**Different parameters**	**Control**	**Low dose**	**High dose**
Fertilization oocyte (%)	88.34 ± 0.88 ^a^	74.66 ± 3.53^b^	48.00 ± 3.20^b^
2- Cell embryo (%)	80.33 ± 1.45^a^	66.65 ± 3.84^b^	44.34 ± 5.45^b^
Blastocyst (%)	81.34 ± 2.89^a^	69.00 ± 1.15^b^	40.34 ± 4.18^b^

The different parameters in the control and test groups are analyzed through Tukey’s test. In each row the identical superscript indicates non- significant (P>0.05) difference between parameters

## Discussion

Pyridaben, a pyridazinone derivative, is a new acaricide and insecticide (4). The other compounds which are commonly used as pesticide including atrazine, benomyl, carbaryl, carbofuran, chlorpyrifos, endosulfan, dibromochloropropane (DBCP), 2, 4- dichlrophenoxy acetic acid, dimethoate, dioxin, hexachlorocyclohexane (HCH), lindane, malathione, mancozeb, methoxychlor, methyl parathion, organophosphate, imidaclopride and pyrethyroid. The reproductive toxic effects of these compounds are widely studied (27, 28). 

At present pyridaben is widely used as miticide and insecticide through all over the world, although this compound is toxic for different biological systems (4, 23, 29). Its noxious effects on the male and female reproductive systems are ill known. In the present study we showed that in pyridaben groups (high and low dose) sperm protamine- histone transition impairments, DNA missed integrity, and consequentially loss of motility increases. Ultimately our results showed that the quality of sperm content become lower and exhibit lower in vitro fertilization rate. In present study we used the effect of various pyridaben doses on maturation and DNA integrity and to identify in vitro fertilization ability of these sperms in different groups.

In the current study after 45 days, pyridaben group showed high morphologically immature sperms. A numbers of studies have suggested that the presence of spermatozoa with damaged DNA may be the result of an impaired chromatin packing or may be indicative of apoptosis (30, 31). Protamines may act as protective elements by sequestering metals capable of promoting the fragmentation of sperm DNA (32). Our special staining for protamine showed that by the time after pyridaben group, the intensive damage affected the sperms protamine-histone transition and this disorder may account in part for the extensive DNA damage observed in poorly packaged spermatozoa of the pyridaben group. 

On the other hand the results from acridine-orange stainings were in good accordance with these findings and showed an elevated sperm DNA fragmentation and damage pyridaben rats. There are reports indicating that any disorder which resulted in a failure in epididymal sperm maturation, causes impaired sperm fertilizing ability (31, 33, 34). Development of spermatozoal ability to expose forward motility, undergoing capacitation, and penetrate the zona pellucida of the oocyte is examples of the several important properties, which the spermatozoa acquire during epididymal sperm passage (1). Our observations revealed that in pyribaden group morphologically immature sperms increased remarkably and the results from IVF correlated reversely with these findings. The ability of the embryo to survive appears to be negatively correlated with the level of DNA fragmentation in the germ line (35). 

Previous studies showed that DNA-damaged sperms cannot fertilize the oocyte (31, 32). Our results in this study showed that by using the sperms from pyribaden rats some of the fertilized oocytes stopped to continue division in two cells and blactocyst embryo phase. Moreover, fertilization rate in treatment group compared with the control group was found remarkably low. 

## Conclusion

These results demonstrated that pyridaben is able to induce DNA damage and chromatin abnormalities in spermatozoa which could be contributed in observed low fertilizations rate.
